# Performance of urinary liver-type fatty acid-binding protein in diabetic nephropathy: A meta-analysis

**DOI:** 10.3389/fmed.2022.914587

**Published:** 2022-09-02

**Authors:** Li Zhang, Shuai Xue, Meiyan Wu, Dan Dong

**Affiliations:** ^1^Department of Nephrology, The First Hospital of Jilin University, Changchun, China; ^2^Thyroid Surgery Department, General Surgery Center, The First Hospital of Jilin University, Changchun, China

**Keywords:** urinary liver-type fatty acid binding protein, diabetic kidney disease, biomarkers, meta-analysis, chronic kidney disease (CKD)

## Abstract

**Aims:**

Diabetic nephropathy (DN) is one of the main causes of chronic kidney disease (CKD), which increases the risk of cardiovascular diseases and progresses to end-stage renal failure. Thus, early diagnostic markers for diabetic patients are urgently needed to improve the prognosis of DN and predict DN progression.

**Materials and methods:**

PubMed, MEDLINE, EMBASE, and Scopus were searched for publications until February 24, 2021. Review Manager 5.4 software was used for meta-analysis. We performed the heterogeneity test using the I^2^ statistic: *P* < 0.1 and I^2^> 50% meant statistical significance.

**Results:**

We included 13 studies. The urinary liver-type fatty acid-binding protein (uL-FABP) concentrations in the normal albuminuria group were significantly higher than those in the normal control group without diabetes mellitus (DM) [*P* = 0.009, SMD 1.72, 95% CI (0.44, 2.99)]. Urinary F-LABP levels were elevated in the macroalbuminuria group compared with those in the microalbuminuria group with DM [*P* = 0.002, SMD 2.82, 95% CI (1.03, 4.61)]. Urinary L-FABP levels were also significantly increased in the progression and CKD groups compared with non-progression and CKD subjects with DM [*P* = 0.02, *P* < 0.00001, respectively]. Furthermore, uL-FABP concentrations were positively correlated with the albumin-to-creatinine ratio and systolic blood pressure in patients with DM [Summary Fisher’s *Z* = 0.58 *P* < 0.00001; Summary Fisher’s *Z* = 0.24 *P* < 0.0001, respectively] and negatively correlated with estimated glomerular filtration rate in patients with DM [Summary Fisher’s *Z* = −0.36, *P* < 0.0001].

**Conclusion:**

Urinary L-FABP may be a potential marker for the detection of all stages of DN and for the prediction of the progression and severity of DN in patients with type 1 and 2 DM.

## Introduction

Diabetic nephropathy (DN) is one of the most common causes of chronic kidney disease (CKD), which increases the risk of cardiovascular diseases and is the leading cause of end-stage renal failure (1–3). Thus, early diagnostic markers for diabetic patients are urgently needed to improve the prognosis of DN and predict DN progression, which is important to initiate appropriate management and treatment in a timely manner (4). Albuminuria or the urinary albumin-to-creatinine ratio (ACR) have been regarded as the standard markers for early detection of DN (5, 6). However, some diabetic patients with persistent microalbuminuria still progress to late stages of CKD (7). Therefore, it is necessary to identify new biomarkers with higher specificity and sensitivity for effective detection and intervention in the pathogenesis of DN to prevent the progression of CKD (8).

Fatty acid-binding protein (FABP) was first discovered in the 1970s by Ockner et al. (9). FABP can bind to long-chain fatty acids and certain other lipids in various tissues including the mammalian adipose tissue, intestinal mucosa, muscle, myocardium, liver, and kidney (9). The liver-type fatty acid-binding protein (L-FABP) is expressed abundantly in hepatocytes and in the convoluted and straight regions of the proximal tubules in humans (10). Recently several studies have shown that L-FABP reflects the oxidative stress level for the progression of different kidney diseases and plays a crucial part in kidney injury and repair especially in renal tubules (11–13). Furthermore, several clinical studies have reported that this protein was elevated in early stages of DN, and predicted that it may become a promising marker for diabetic kidney diseases (4, 10, 14–16). However, no causal clinical correlations have been certified.

As far as we know, there was no meta-analysis has been conducted to investigate the performance of uL-FABP in DN patients till now, although there have been a dozen of studies on the correlation between uL-FABP and diabetic nephropathy. Hence, we did a meta-analysis to synthesize available evidence and explore the performance of uL-FABP in patients with DM.

## Materials and methods

This meta-analysis was designed and guided based on Systematic Reviews guidelines in the Cochrane Handbook (17).

### Literature search

PubMed, MEDLINE, EMBASE, and Scopus were searched for publications until February 24, 2021, without any language limits. Medical Subject Headings key words including “diabetic kidney disease,” “diabetic nephropathy,” “L-FABP OR liver-type fatty Acid-binding protein,” “predictor*,” “biomarker*,” “correlated OR correlation” were used when searching the databases.

### Study selection

Patients: subjects with age over than eighteen years who had been diagnosed with type 1 or type 2 diabetes mellitus (T1DM or T2DM) on the basis of the criteria of World Health Organization (18);

Intervention: DM patients with albuminuria or CKD; normal albuminuria referred to the value of albumin-to-creatinine ratio (ACR) < 30 μg/mg, microalbuminuria indicated an ACR of 30–299 μg/mg, whereas macroalbuminuria referred to ACR ≥ 300 μg/mg (19). The estimated glomerular filtration rate (eGFR) was calculated by using the Modification of Diet in Renal Disease Formula (MDRD-GFR) (20);

Outcomes: uL-FABP concentrations comparisons or correlation analysis of uL-FABP and clinical indexes such as eGFR, HbA1c, ACR;

Study designs: cross-sectional study, case-control trial, longitudinal study or randomized controlled trial.

### Data extraction

We imported all the searched results into the EndNote software (Clarivate Analytics). We removed duplicate publications by EndNote software or manual checking. These abstracts of the remaining publications were checked by L.Z. and M.W. independently for relevance against the inclusion and exclusion criteria. If there was any uncertainty regarding the records, the full texts were retrieved to be checked in detail. If the included data is incomplete, we will contact the author by email to request the data. Any disagreements were discussed with a third reviewer. The risk of bias of the included studies was evaluated by using the Newcastle-Ottawa Scale (NOS) (21). The risk of bias assessment was confirmed independently by L.Z. and M.W.

### Statistical analysis

Review Manager (RevMan) 5.4 and Excel softwares were used for this study. Continuous data were obtained by calculating the standardized mean differences (SMDs) with 95% confidence intervals (CIs) if different scales were applied. We performed the heterogeneity test using the I^2^ statistic across studies, when P < 0.1 and I^2^> 50% statistical significance was indicated. If heterogeneity was not statistically significant, a fixed-effects model was used; otherwise we chose a random-effects model (22). If there was significant heterogeneity, we did sensitivity analysis or/and subgroup analysis. Sensitivity analysis was conducted to check every trial’s influence on the pooled results.

Because the correlation coefficient r does not follow normal distribution, Fisher proposed the “Fisher Z Transform” when *r* > 0.5, which transformed the correlation coefficient r into the normal distribution variable Z (23) according to these formulas:


(1) $$\text{Fisher's }Z\;=\;0.5*\text{ln}\frac{1+\text{r}}{1-\text{r}}$$

(2) $$S\,E=\sqrt{1/\left(n-3\right)}\,n\,\mathrm{is}\,\mathrm{the}\,\mathrm{sample}\,\mathrm{size}$$

(3) $$\mathrm{Summary}\,r=\frac{{\text{e}}^{2\,Z}-1}{{\text{e}}^{2\,Z}+1}$$

We converted data by using Excel. Formula (1) and (2) were used for obtaining Fisher’s Z and standard error (SE). Then, we used the combined effect value of the correlation coefficient r to evaluate the strength of the correlation by using formula (3). Finally, the summary *r* was used to judge the strength of correlation: ≥ 0.8 high correlation, 0.3-0.8 moderate correlation, and ≤0.3 indicated low correlation (23).

## Results

### Search results

**FIGURE 1:**
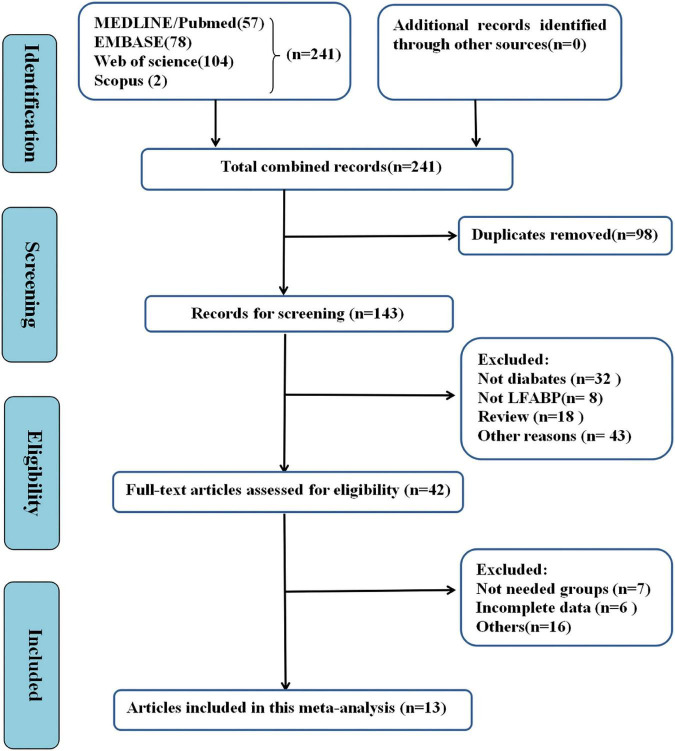
Flow chart of screening process.

We identified 239 articles throughout databases of PubMed, MEDLINE, and EMBASE. Exclusion of duplicate records and screening of the abstracts or full texts yielded 41 articles. At last, we included 13 studies as illustrated in Figure 1.

### Study characteristics

A total of 5,605 participants was enrolled in the study. The characteristics of included studies were shown in Table 1. Four studies were conducted on subjects with T1DM (16, 24–26) and nine studies on T2DM subjects (6, 27–34). Four studies were conducted in Japan (30–33), and the other studies were in Egypt (16), Denmark (24), Australia (25), South Korea (26), Taiwan/China (27), Vietnam (6), Germany (28), India (34), and the Gila River Indian Community (29). NOS scores for quality assessment were shown in Table 1.

**TABLE 1:** Characteristics of the included studies.

Author	Year	Country/Region	Study Design	DM type	Sample size	Sex (Male/Female)	Age (year, mean ± SD or median (range))	Method	NOS
Abd El Dayem (16)	2015	Egypt	cross-sectional	1	92	DM: 31/31	16.32 ± 1.52	ELISA	6
						C: 15/15	16.3 ± 2.63		
Nielsen (24)	2010	Denmark	cross-sectional	1	204	118/86	38 ± 12.6	ELISA	8
Panduru (25)	2013	Australia	cross-sectional	1	2454	DM: 1126/1120	NA	ELISA	8
						C: 106/102	35.9 ± 11.3		
Suh (26)	2016	South Korea	cross-sectional	1	61	DM: 12/17	NA	ELISA	7
						C: 13/19	11.91 ± 3.61		
Chou (27)	2013	Taiwan/China	longitudinal	2	140	72/68	56.6 ± 9.8	ELISA	6
Thi (6)	2020	Vietnam	cross-sectional	2	136	60/76	NA	ELISA	7
Eynatten (28)	2010	Germany	cross-sectional	2	170	125/45	DM:60.7 ± 7.4	ELISA	8
							C:51.9 ± 9.5		
Fufaa (29)	2015	the Gila River Indian Community	longitudinal	2	260	82/178	42.5(18.7-65.1)	ELISA	8
Gohda (30)	2018	Japan	cross-sectional	2	314	166/148	64 ± 13	ELISA	7
Ito (31)	2017	Japan	cross-sectional	2	788	457/331	66 ± 12	ELISA	6
Kamijo (32)	2011	Japan	cross-sectional	2	552	88/52	NA	ELISA	7
Suzuki (33)	2005	Japan	cross-sectional	2	356	229/127	63 ± 11	ELISA	7
Viswanathan (34)	2015	India	cross-sectional	2	78	45/33	NA	ELISA	7

DM, diabetes mellitus; C, control; NOS, Newcastle-Ottawa Scale; NA, Not Available; ELISA, enzyme linked immunosorbent assay.

### Albuminuria in DM

**FIGURE 2:**
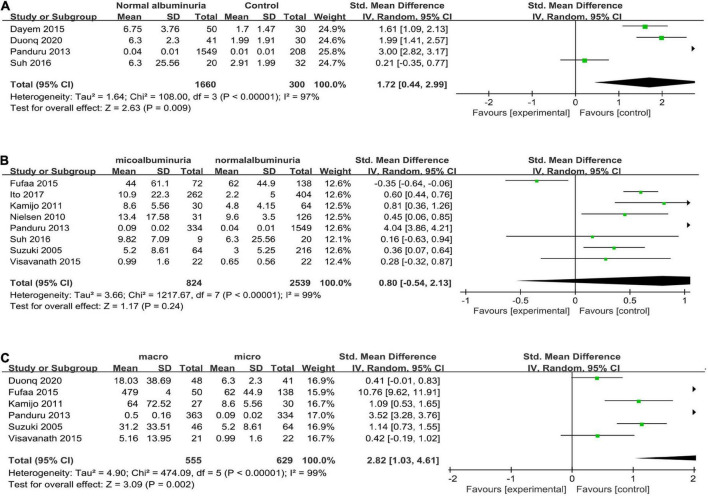
**(A)** Forest plot of u-LFABP level comparison in normal control group and diabetic patients with normal albuminuria group; **(B)** Forest plot of u-LFABP level comparison in diabetic patients with micro albuminuria group and normal albuminuria group; **(C)** Forest plot of u-LFABP level comparison in diabetic patients with macro albuminuria group and micro albuminuria group.

Four articles (16, 25, 26, 29) reported uL-FABP values for the normal albuminuria DM group and normal control subjects without DM. Because of different scales of the uL-FABP concentrations, SMD was selected. The uL-FABP values of the normal albuminuria group with DM were significantly elevated than those in the control group without DM [*P* = 0.009, SMD 1.72, 95% CI (0.44, 2.99)] but showed obvious heterogeneity (Figure 2A).

Eight trials (24–26, 29, 31–34), including 3363 participants, reported uL-FABP concentrations in the normal albuminuria and microalbuminuria groups. The results presented no significant difference exists in the uL-FABP concentrations between the normal albuminuria and the microalbuminuria groups of DM patients [*P* = 0.24] (Figure 2B). Six studies (6, 25, 29, 32–34), including 1184 participants, reported uL-FABP concentrations in the macroalbuminuria and microalbuminuria groups with DM. Moreover, uL-FABP concentrations were significantly elevated in macroalbuminuria group compared to microalbuminuria group among DM patients [*P* = 0.002, SMD 2.82, 95% CI (1.03, 4.61)] (Figure 2C). However, there was significant heterogeneity.

### Progressive diabetic nephropathy

**FIGURE 3:**
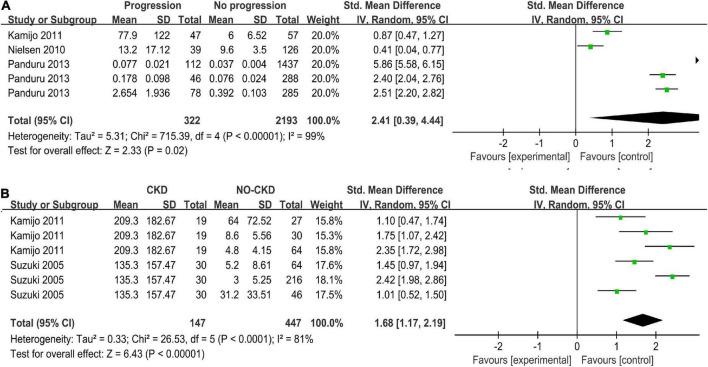
**(A)** Meta-analysis Forest plots of u-LFABP concentrations comparison in progressive DM group and non-progressive DM group; **(B)** Meta-analysis Forest plots of u-LFABP concentrations comparison in DM with CKD and DM without CKD.

The progression group was defined as patients whose DN had developed from one stage to the next stage such as microalbuminuria, macroalbuminuria, end-stage renal failure, or induction of hemodialysis (24, 25, 32). Three studies with five arms compared uL-FABP concentrations in progression and non-progression groups (24, 25, 32). The results showed that uL-FABP concentrations were significantly higher in the progression group than in the non-progression group [*P* = 0.02, SMD 2.41, 95% CI (0.39, 4.44)] (Figure 3A).

### Chronic kidney disease

Two studies with six arms reported uL-FABP concentrations in CKD group and without CKD group among DM patients (32, 33). The uL-FABP concentrations in the DM with CKD group were significantly elevated compared with those in the DM group without CKD [Figures 3B, *P* < 0.00001, SMD 1.68, 95% CI (1.17, 2.19)].

### Correlation analysis between uL-fABP and clinical indexes

To explore the relationships between uL-FABP concentration and DN, we performed a correlation analysis between uL-FABP and ACR, eGFR, SBP, HbA1c, and FPG.

### uL-FABP and albumin-to-creatinine ratio

**FIGURE 4:**
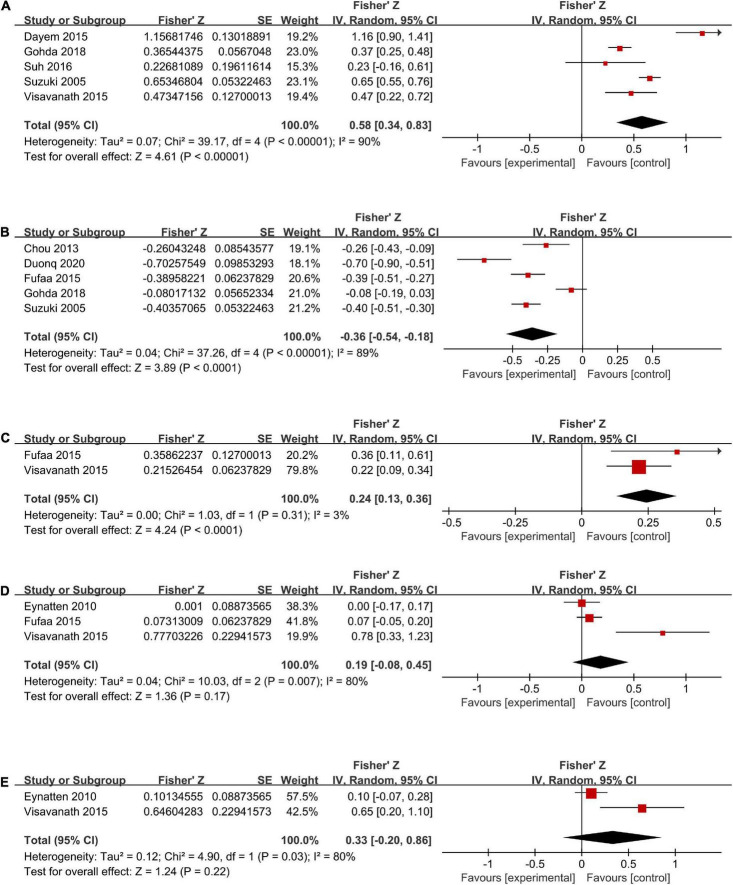
Forest plots of correlation analysis between u-LFABP values and different clinical index of ACR **(A)**, eGFR **(B)**, SBP **(C)**, HbA1c **(D)**, FPG **(E)**.

Five studies (6, 26, 30, 33, 34) (total n = 826) investigated the correlation between the levels of uL-FABP and ACR (Figure 4A). It was showed that ACR was positively correlated with uL-FABP values in diabetic patients [Summary Fisher’s *Z* = 0.58, 95% CI (0.34, 0.83), *P* < 0.00001]. The summary r indicated moderate correlation.

### uL-FABP and estimated glomerular filtration rate

Five trials (6, 27, 29, 30, 33) (total *n* = 1176) had investigated the correlation between uL-FABP levels and eGFR. The results showed that uL-FABP levels were negatively correlated with eGFR in diabetic patients [Summary Fisher’s *Z* = −0.36, 95% CI (−0.54, −0.18), *P* < 0.0001]. The final summary r indicated moderate correlation (Figure 4B).

### uL-FABP and systolic blood pressure

Two trials (29, 34) had performed correlation analysis between uL-FABP levels and SBP. We found that uL-FABP was positively correlated with SBP in patients with DM [Summary Fisher’s *Z* = 0.24, 95% CI (0.13,0.36), *P* < 0.0001]. However, summary r value was only 0.24, indicating low correlation (Figure 4C).

### uL-FABP and glycosylated hemoglobin, fasting plasma glucose

Three articles (28, 29, 34) had conducted correlation analysis between uL-FABP levels and HbA1c (total *n* = 412; Figure 4D), and two articles (28, 34) had analyzed the correlation between uL-FABP levels and FPG (total *n* = 325; Figure 4E). We found that uL-FABP levels were not significantly correlated with HbA1c or FPG. [*P* = 0.17, *P* = 0.22, respectively].

### Subgroup analysis

**FIGURE 5:**
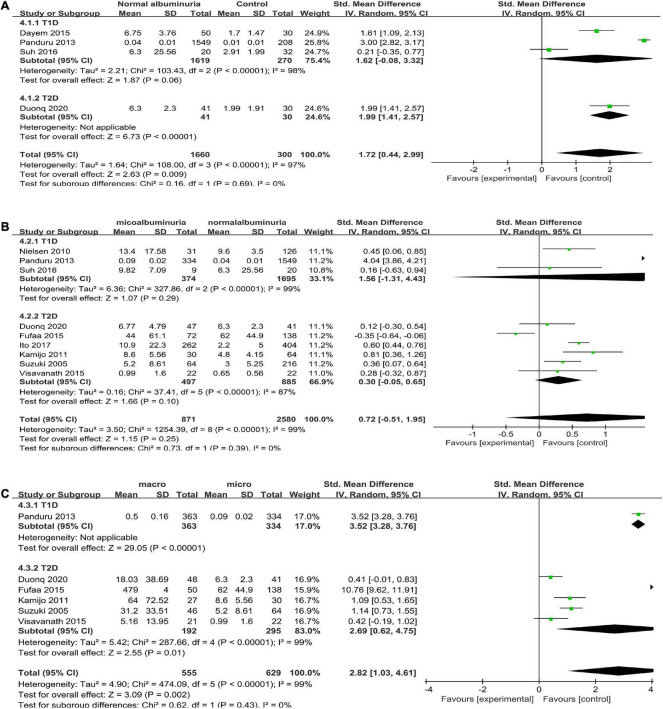
Subgroup analysis of different albuminuria groups in DM according to diabetes types **(A)** Normal albuminuria group vs. Normal control group; **(B)** Miro albuminuria group vs. Normal albuminuria group; **(C)** Macro albuminuria group vs. Micro albuminuria group.

**FIGURE 6:**
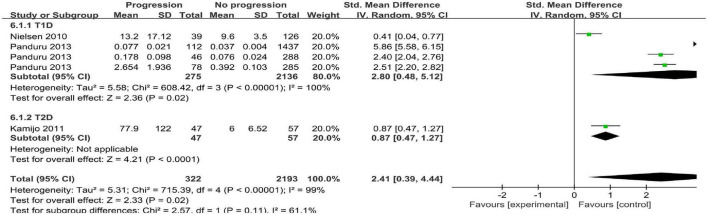
Subgroup analysis for DM with progressive group.

As there was all significant heterogeneity in all the analysis, we conducted subgroup analysis according to the type of diabetes. As the comparison of DM with CKD and non-CKD groups was performed only in type 2 DM subjects, subgroups were not formed. We did not find any decline in the heterogeneity (Figures 5, 6). Moreover, all the results of different albuminuria comparisons and progression in T1D and T2D subjects were similar to the combined results.

### Sensitivity analysis

We also conducted leave-one-out sensitive analysis to explore the potential source of heterogeneity. In T1D subgroup analysis of microalbuminuria and normal albuminuria comparison, I^2^ of heterogeneity was reduced to 0% after excluding the study conducted by Panduru et al. (25). Similarly, in the T2D subgroup analysis of microalbuminuria and normal albuminuria comparison, I^2^ of heterogeneity was reduced to 50% from 87% after excluding Fufaa et al’s study (29). Thus, we found among diabetic patients uL-FABP levels of the microalbumuria group were increased significantly than in the normal albuminuria group after excluding the above studies, without any obvious heterogeneity (*P* = 0.03, *P* < 0.0001, respectively, Supplementary Figure 1). In other sensitivity analysis, I^2^ of heterogeneity was not reduced sharply. However, after excluding each study, the results were not significantly changed, indicating that the combined results were steady.

## Discussion

So far, this may be the first meta-analysis on the associations between uL-FABP levels and kidney injury in patients with DM. We found that uF-LABP levels were significantly elevated in the normal albuminuria DM group compared with the control group without DM. Urinary L-FABP could be a potential biomarker of tubulointerstitial injury (12). Tanaka et al. showed there was a positive correlation between uL-FABP and pathological injury of fibrosis and macrophage infiltration in animals (12). However, high uL-FABP levels in normoalbuminuric patients may indicate that tubulointerstitial damage occurred prior to the glomerular injury in subjects with DM (35). Thus, uL-FABP may be an earlier indicator than ACR or urinary albumin in the detection of renal injury in subjects with DM (6). Furthermore, >30% of diabetic patients with normal albuminuria were considered to have histological kidney injury (36). Similarly, we found compared with microalbuminuria group uF-LABP levels were elevated in macroalbuminuria groups in the subjects with DM. After observing declining heterogeneity in subgroup and sensitive analyses, we found that uL-FABP levels were elevated in microalbuminuria groups compared with those in the normal albuminuria group among the subjects with T1D and T2D. Moreover, a strong correlation between uL-FABP and ACR was reported by the analysis. Panduru et al. postulated that after microalbuminuria appears, the increase in fatty acid binding to albumin may lead to fatty acid overload in the proximal tubules due to which the *L-FABP* gene may be up-regulated to increase free fatty acid export into the mitochondria (25). This hypothesis was considered controversial (13, 37, 38).

Moreover, the current study revealed that the uL-FABP level was significantly increased in the progression group of subjects with DM compared with the non-progression DM group. Further, we found that the uL-FABP level was significantly increased in the CKD group of subjects with DM compared to the non-CKD group of subjects with DM. Urinary L-FABP also shows a strong correlation with eGFR in our analyses. Several studies have shown that renal tubular damage plays a key role in the pathogenesis of DN (39–41). Ishimitsu et al. found there was a close correlation between the peritubular blood flow and uL-FABP, indicating that uL-FABP was a sensitive marker for microcirculation dysfunction in the proximal tubules of the kidney (35). Along with the worsening of renal damage in DN, there is gradual aggravation of tubular injury (29). In the late stages of DN, hypoxia and oxidative stress probably cooperate with the increased albuminuria excretion, resulting in the elevation of uL-FABP (42). Although, in experimental models of CKD, uL-FABP has been confirmed to be correlated with fibrotic changes (12), uL-FABP was considered a potential marker of early detection for acute kidney injury in the meta-analysis by Susantitaphong et al., albeit with scanty evidence (43). Tubular markers were always thought to be associated with the progression of glomerular diseases when acute tubular necrosis accompanied diabetic glomerular disease (29). Sharma et al. concluded there was a significant correlation between acute tubular necrosis and diabetic glomerulosclerosis (44). Therefore, we postulate that uL-FABP could be a sensitive marker, not only for the early diagnosis of DN, but also for estimating the progression and severity of DN (32).

Interestingly, a correlation was reported between SBP and uL-FABP in subjects with DM in this meta-analysis. This is consistent with the findings reported by Okubo et al. (45), which showed that elevated uL-FABP levels may predict renal dysfunction progression and cardiovascular adverse events even among non-diabetic subjects with hypertension (45). Glomerularsclerosis and activation of the renin–angiotensin system lead to decreased functional flow to the proximal tubules and cause anoxia in the tubules, leading to uL-FABP excretion in patients with hypertension, with or without DM (46). Nevertheless, we did not find any significant correlation between uL-FABP and HbA1c or FPG. However, Ito et al. speculated that cytotoxic factors, such as energy deficiency in the proximal tubular cells that resulted from hyperglycemia, might have induced uL-FABP excretion (31). However, few clinical studies have explored the relationship between uL-FABP values and blood glucose levels. Thus, more long-term clinical data are required to verify these findings.

The present meta-analysis included the latest clinical trials and may be the first to investigate the role of uL-FABP in subjects with diabetes. We included 13 studies of good quality. However, the present study has some limitations. On the one hand, the heterogeneity of most results in this study was significant. But the sensitivity analysis showed that the final consequences were stable. The possible reasons may be that the uL-FABP concentrations had been measured by different reagent kits in different trials, due to which the diagnostic thresholds were also different. In addition, the inclusion criteria of each study were different from each other. On the other hand, the study types of the included studies were mostly cross-sectional with small samples. To explore the performance of uL-FABP levels on diabetic patients, we need more prospective longitudinal studies on patients with T1DM and T2DM, with more samples and longer study duration.

In summary, uL-FABP may be a potential marker for the detection of all stages of DN and for the prediction of the progression and severity of DN in the patients with T1DM and T2DM. In addition, uL-FABP was a useful biomarker for the early detection of kidney injury, even when urinary albumin levels were in the normal range.

## Data availability statement

The original contributions presented in this study are included in the article/Supplementary material, further inquiries can be directed to the corresponding author/s.

## Author contributions

LZ and MW searched for the articles and assessed the search results. Any disagreement was resolved through discussions or consultations with SX. LZ evaluated the risk of bias in each included study. MW checked the risk of bias in the assessment. LZ and DD wrote the manuscript. SX was responsible for valuable intellectual content during the revision of the manuscript. All authors issued a final approval for the submitted version.
