# Supplementary figures and images for: Performance of urinary liver-type fatty acid-binding protein in diabetic nephropathy: A meta-analysis
Source: Front Med (Lausanne). 2022 Sep 2;9:914587. doi: 10.3389/fmed.2022.914587 (PMC9479543; doi:10.3389/fmed.2022.914587)

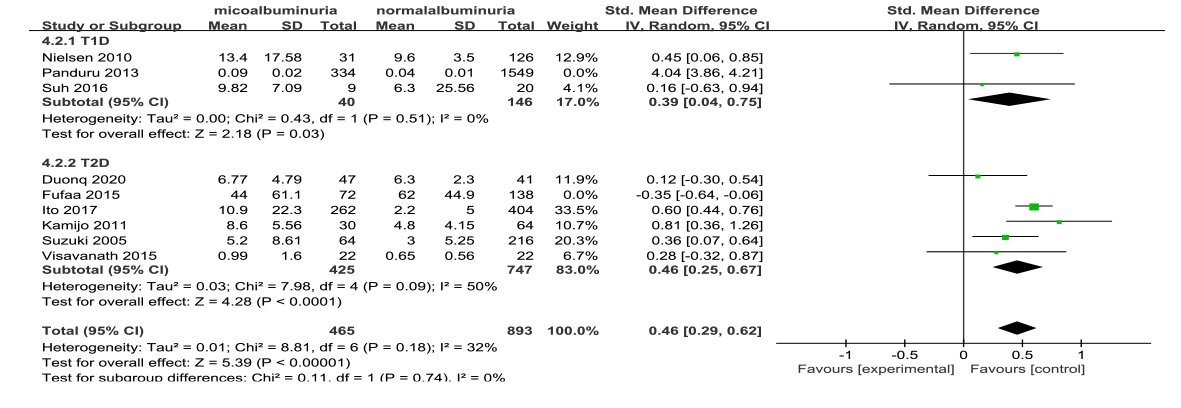


FIGURE S1. Sensitivity analysis.

Supplement: Supplementary file 1 [file Data_Sheet_1.doc]
